# Simulation of performance evaluation model for medical-elderly care integrated institutions based on system dynamics

**DOI:** 10.1186/s12913-022-08835-0

**Published:** 2022-11-30

**Authors:** Yongqiang Shi, Fangfang Fan, Zhiyong Zhang

**Affiliations:** Department of Electronic Business, South China University of Technology, No. 382, Waihuan East Road, Xiaoguwei Street, Panyu District, Guangzhou, Guangdong 511400 People’s Republic of China

**Keywords:** Medical-care integration models, Performance evaluation, System dynamics, Sensitivity analysis

## Abstract

**Background:**

Based on the Chinese model of medical-elderly care integration, this paper aims to explore the impact of different investment levels on the performance of the medical-elderly care integrated institutions.

**Methods:**

Using the method of system dynamics, this paper establishes the performance evaluation model of medical-elderly care integrated institutions, sets the system element input, service level, and policy support as the key factors, and uses Vensim PLE software for simulation.

**Results:**

The three key factors have different degrees of positive impact on the performance of medical-elderly care integrated institutions. On the whole, policy support has the most significant impact on the performance of institutions, followed by the level of medical-elderly care integrated services. Institutional input mainly has a great impact on the performance of institutions in the early stage. In addition, the model simulation results also show the emergence effect: the improvement rate of institutional performance under the comprehensive simulation is higher than the sum of the improvement rates under the separate action of single factor.

**Conclusion:**

Government policies have played an important role in promoting the development of medical-elderly care integrated institutions. The service level and resource input can effectively promote the performance of medical-elderly care integrated institutions. Institutions should formulate development strategies from a systematic perspective, and pay attention to the integration of "medical" and "elderly care" resources.

## Introduction

According to the results of China's seventh census in 2020, China's population aged 60 and above has exceeded 264 million, accounting for nearly 1/5 of the total population. China is about to enter a moderately aging society. At the same time, among China's huge elderly population, the proportion of the elderly suffering from chronic diseases has exceeded 3/4 [1]. Traditional elderly care institutions only provide a single daily care service, which can no longer meet the medical needs of the elderly [2]. The phenomenon of "difficult and expensive medical treatment" in medical institutions is particularly serious among the elderly population. The development of medical-elderly care integrated institutions has attracted more and more attention from all sectors of society [3, 4].

The international community has paid attention to the medical needs of the elderly earlier and proposed "integrated elderly care services". In 2012, the World Health Organization defined it as "Integrating the investment, delivery, management and organization of services related to diagnosis, treatment, care, rehabilitation and health promotion" [5]. At present, the relatively mature models that combine medical care and elderly care to provide services include the PACE model in the United States [6–8], the integrated care model in the United Kingdom [9, 10], and the PRISMA model in Canada [11, 12]. These models provide medical and health services to the elderly in different ways, focusing on the clinical effect of the medical team on the elderly in the nursing home [13]. Although the elderly care model in some developed countries has been improved and perfected, it may still face the risk of "acclimatization" in China [14]. Therefore, in 2005, China first introduced the concept of "Integration of medical care and elderly care, continuous care". Starting from the diversified needs of the elderly, through the organic integration of elderly care and medical resources, provide the elderly with a series of more comprehensive services in addition to basic daily life care, such as medical treatment, rehabilitation, health education, etc. [15]. The medical-elderly care integrated services in China pay more attention to the integration of medical and elderly care resources. At present, it is mainly developed into three modes: setting up elderly care in medical institutions, setting up medical services in elderly care institutions, and cooperation between medical and elderly care institutions. Setting up elderly care in medical institutions means adding elderly care services in medical institutions. Medical institutions can provide professional long-term care services for the elderly and can make full use of medical resources. Setting up medical services in elderly care institutions means that the elderly care institutions can provide basic medical services by configuring medical offices, nursing stations, and outpatient departments. In addition to providing basic elderly care services, this mode can also enhance the ability of medical security and meet the needs of the elderly for disease treatment and rehabilitation care. The third type is cooperation between medical and elderly care institutions, that is, elderly care institutions and medical institutions jointly provide services by signing contract. The medical institutions include the elderly in health management and provide convenience for the elderly to seek medical treatment. Doctors can also visit elderly care institutions regularly [16]. In China, the model of medical and elderly care cooperation is the most common. It can enable elderly care institutions and medical institutions to form a two-way care model of benign interaction, make full use of the resources of medical institutions and elderly care institutions, and complement each other's advantages. In this paper, the analysis of the performance of medical-elderly care integrated institutions is also mainly based on this model.

In 2013, the State Council of the People's Republic of China officially clarified the development concept of "Integration of medical care and elderly care". Since then, the medical-elderly care integrated institutions have entered a stage of rapid development. By the end of 2020, more than 90% percent of elderly care institutions in China can provide different forms of medical services for the elderly. The medical-elderly care integrated institutions play an important role in integrating medical and elderly care resources, improving the happiness and satisfaction of the elderly, and helping the development of healthy aging. However, due to China’s basic national conditions of "Getting old before getting rich" and the late start of the elderly care industry, there are many problems such as the separation of medical and elderly care and the imperfect evaluation system of services in the medical-elderly care integrated institutions [17, 18], which makes it difficult to ensure the quality of elderly care services. The results of the performance evaluation of the medical-elderly care integrated institutions are directly related to the quality of elderly care services and the development of elderly care institutions in China [19, 20].In this context, this paper uses the system dynamics(SD) method to analyze the performance of medical-elderly care integrated institutions, and quantitatively explores the impact of changes in key factors such as institutional resource input and policy support on the performance of the institutions, to enrich the connotation and research methods of service performance evaluation of Chinese medical-elderly care integrated institutions, provide a reference for the formulation of government policies, and provide directions for the transformation of traditional elderly care institutions to the mode of medical-elderly care integration.

## Methods

### Applicability analysis of SD model

SD is a science that combines system management science with computer simulation and has feedback structure and dynamic reflection. It can simplify the actual operating conditions of the research object and provide operable information to decision-makers concisely. Generally, the application of SD requires the system to have certain characteristics and conditions, such as clear boundary, dynamic law, and predictability [21]. In the performance evaluation system of medical-elderly care integrated institutions, there is a close relationship between the influencing factors and the institutional performance, and the system boundary is clear. The changes of some factors may dynamically affect the performance of the institutions. In addition, through the collection of extensive literature, it is found that the academic circles have very mature experience in the research of institutional performance using SD. Bianchi et al. found that SD can help decision-makers explore how to rapidly improve efficiency and improve results under existing constraints by analyzing three department performance management cases. This conclusion shows that SD can play a positive role in improving department performance [22]. Later, Mudhafar and colleagues identified key factors of employee performance and built an SD model of employee performance, which proves that SD has a good effect in evaluating the impact of different improvement measures on employee performance [23]. Some scholars also use the SD method to develop performance management models to provide managers with visual action feedback [24, 25]. At the same time, the SD method also has incomparable advantages in assisting managers to make decisions. Policy decisions are abstract and complex, and the system dynamics method can provide an accessible basis for policy-making from the endogenous perspective of model simulation [26]. Yuan et al. found that the SD method can dynamically integrate all basic activities in the system by analyzing the benefits of building demolition in Shenzhen, China, and help decision-makers understand which measures can increase net income [27]. Xiong and colleagues designed a system dynamics feedback loop for project cooperation, and the results show that the satisfaction adjustment model based on system dynamics can well improve the overall performance of the project [28]. Therefore, this paper believes that the SD method is applicable to the study of the relationship between influencing factors and the performance of medical-elderly care integrated institutions.

### Factors influencing the performance of medical-elderly care integrated institutions

The performance of medical-elderly care integrated institutions is the product of the comprehensive action of many factors. At present, there is no unified standard for it in China. By sorting out the existing literature, it is found that the performance of medical-elderly care integrated institutions is mainly concentrated in three endogenous variables: Institutional input, Service output, and Service effect, and one exogenous variable: Policy support. Table 1 lists the 13 performance evaluation indicators of medical-elderly care integrated institutions most concerned by the academic community since 2011. These researches provide a necessary foundation for further in-depth analysis of the performance of medical-elderly care integrated institutions.

**Table 1:** Factors influencing the performance of medical-elderly care integrated institutions

Primary index	Secondary index	Supporting literature
Institutional input	Information system resource investment	[16, 20, 29–32]
	Human investment	[18, 20, 29, 31–43]
	Facility and equipment investment	[18, 20, 29, 31–33, 35–39, 41–43]
	Capital investment	[20, 32, 36–39, 42, 44, 45]
Service output	Service sustainability	[38, 46, 47]
	Service efficiency	[37, 38]
	Service content	[18, 20, 30, 32, 34, 35, 39–42, 46]
	Service equity	[37, 38, 46]
	Service capability	[16, 18, 20, 29, 30, 33, 34, 36, 39–41, 44, 45]
	Service quality	[18, 20, 31, 32, 37]
Service effect	Satisfaction of the relevant personnel	[18, 20, 31, 32, 38, 40, 41, 44–46]
	Social recognition of institutions	[18, 20, 29, 32, 35, 42]
Exogenous variable	Policy support	[16, 31–33, 45, 48, 49]

### Subsystem analysis

Taking the institutional performance as the breakthrough, and according to the correlation and subordination between indicators, the indicators are combined to form a multi-level performance evaluation system of medical-elderly care integrated institutions, which includes three subsystems: Institutional input, Service output, and Institutional satisfaction.

A. Institutional input subsystem, which is the basis of institutional operation. In the institutional performance evaluation system, institutional investment mainly includes human investment, facility and equipment investment, information system resource investment, and capital investment.
B. Service output subsystem, which mainly measures the service level, including service quality, service content, and service sustainability. The services of medical-elderly care integration provided by institutions to the elderly are mainly transformed from institutional input.
C. Institutional satisfaction subsystem, which can be directly reflected by the satisfaction of the relevant personnel of the institutions, or indirectly reflected by the social image of the institutions, and the satisfaction of the relevant personnel includes the satisfaction of staff, the elderly and their families. Table 2 reflects the boundaries and variables of the three subsystems.

**Table 2:** Subsystem and its variables

Subsystem	Main variables
Institutional input	Information system resource investment, Facility and equipment investment, Capital investment, Institutional profit and social capital investment, Local financial subsidies, Financial state of the institution, Medical staff, Administrative staff, Proportion of people holding professional certificates, Proportion of people receiving professional training
Service output	Service content, Service quality, Service capability, Service sustainability, Operation management, Cooperation mechanism of medical and elderly care institutions, Turnover of service staff, Rounds of visits of cooperative medical institutions, Rehabilitation nursing, Health monitoring, Diagnosis and treatment of basic diseases, Long-range medical treatment
Institutional satisfaction	Elderly satisfaction, Family and guardian Satisfaction, Institutional employee Satisfaction, Public satisfaction, Institutional image, Service complaints, Institutional social recognition

**Fig. 1:**
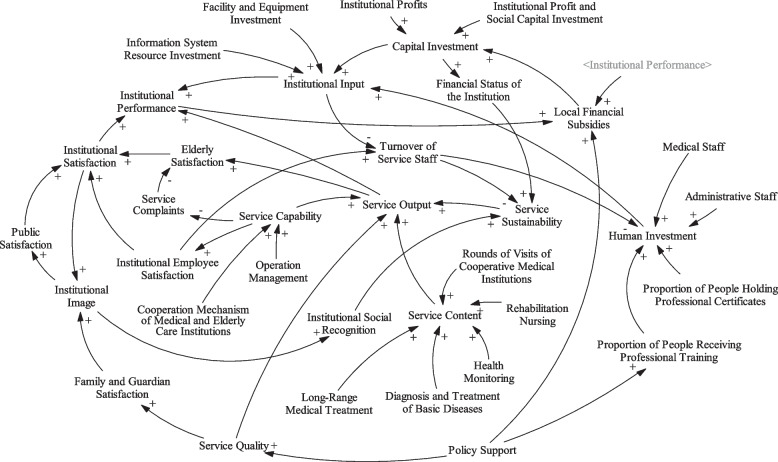
Causality diagram of performance evaluation system

The more institutional investment, the higher the services level of medical-elderly care integration that institutions can provide, and the more satisfaction of the elderly, which is finally reflected in the improvement of institutional performance. Under the condition of a stable development environment of national medical-elderly care integration, the higher the performance means the higher the profitability of the institutions, which determines the next round of investment scale. The causality diagram of performance evaluation of medical-elderly care integrated institutions is constructed (Fig. 1).

### Determination of key factors

The factors that have the most direct impact on institutional performance are identified as key factors. Firstly, these factors have more connections with other variables and can be used as representatives of other variables in the subsystem. Secondly, key factors have the most direct impact on system performance.

**Element input**, which is the cornerstone of institutional development and providing medical-elderly care integrated services for the elderly, has a far-reaching impact on other variables of the system. Element input includes human input and material input.

**Service level**, which has the most direct relationship with the satisfaction of the elderly in institutions. Service level includes service quality and the content of combined medical and elderly services.

**Policy support**, which is the guarantee of the external environment for the development of institutions, usually plays an irreplaceable role in the supply of service talents, government subsidies to institutions, and the quality norms of services [50].

### Construction of SD flow diagram

**Fig. 2:**
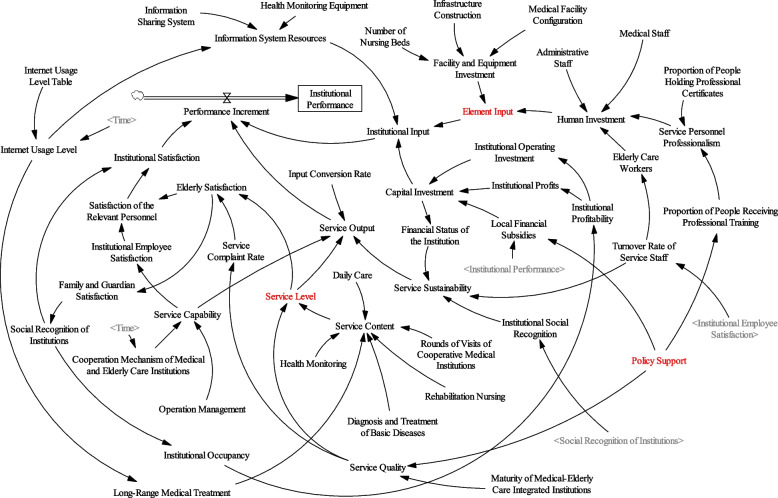
Dynamic flow chart of performance evaluation system

Based on the causality diagram and the feedback between various factors, this paper introduces 2 stock variables, 33 auxiliary variables, and 17 constant variables to construct the dynamic flow chart of the performance evaluation system of medical-elderly care integrated institutions, as shown in Fig. 2.

### Equation construction and parameter setting

For SD equation design and variable assignment, comprehensive estimation, literature review, and experimental adjustment are adopted. The SD equations were developed by drawing on the views of Wang LJ, Zhang LW, and Zhu L, et al. and combined with the actual situation of this study. For variables that are difficult to determine, the optimal value is sought by constantly adjusting parameters during model operation. Because the variable units in the model are not unified, this paper makes it dimensionless, and the value range is [0,1] [51]. The model equations and their explanations are shown in Table 3.

**Table 3:** Design and explanation of the SD equation

Equation design	Explanation
(1) Institutional performance = INTEG (Institutional input + Service output + Institutional satisfaction)	Institutional performance consists of Institutional input, Service output, and Institutional satisfaction. The initial value is set to 1
(2) Institutional input = 0.1* Information system resource investment + 0.5* Element input + 0.2* Capital investment	The weights of the three components of Institutional input are slightly different in the literature. This paper takes the average value of the research results of various scholars
(3) Human Investment = Administrative staff + (Medical staff + Elderly care workers)* Service personnel professionalism	The professional degree of service personnel who directly serve the elderly has a great impact on the level of human resources in institutions
(4) Institutional operating investment = STEP(0.1* Institutional profitability, 36) + 0.5	The initial operating investment of the institution is 0.5, and 10% of the institutional profit begin to be the main investment after three years
(5) Local financial subsidies = Policy support *PULSE TRAIN(12,1,12,120)	Government subsidies to institutions have a time interval, generally once a year
(6) Information system resources = (0.3* Information sharing system + 0.3* Health monitoring equipment)* Internet usage level	The Internet usage level has a positive impact on the use of institutional hardware and software
(7) Service output = (Service capability + Service level + Service sustainability)* Input conversion rate	Considering the depreciation and the turnover, the resource input of the institution cannot be fully transformed into the output of the institution
(8) Input conversion rate = 0.8	It is assumed that 80% of institutional input can be transformed into institutional service output
(9) Service capability = Service content * Service quality	Service capability is affected by service content and service quality
(10) Service quality = SMOOTH(Policy support,60,0.1)* Maturity of medical-elderly care integrated institutions	The role of quality norms is not achieved overnight. It is assumed that the 60th month after the release of the policy can fully play the role of quality assurance
(11) Service sustainability = (Institutional social recognition + Financial Status of the Institution)/ Turnover rate of service staff	The social recognition and financial status of institutions have a positive impact on service sustainability, while the turnover rate has a negative impact
(12) Institutional satisfaction = 0.35* Social recognition of institutions + 0.65* Satisfaction of the relevant personnel	The weight setting method is the same as the Institutional input
(13) Elderly satisfaction = Service level / Service complaint rate	Elderly satisfaction is positively correlated with service level and negatively correlated with service complaint rate
(14) Service complaint rate = 1/ Service quality	The service complaint rate is negatively correlated with service quality
(15) Family and guardian satisfaction = DELAY1(Elderly satisfaction,2)	The evaluation of the institution by the family members directly comes from the elderly, but there is a time difference. The satisfaction of the elderly will affect the family members after two months
(16) Internet usage level = Internet usage level table(Time)	The change of Internet usage level over time is nonlinear, so the model uses a table function
(17) Internet usage level table = [(0,0.7)- (130,0.8)],(0,0.7),(10,0.73),(20,0.76), (30,0.773),(40,0.781),(50,0.785), (60,0.788),(70,0.789),(80,0.79), (90,0.791),(100,0.792),(110,0.793), (120,0.794)	From LJ [52]: Prediction of Internet penetration in China based on innovation diffusion theory

## Result

### Model validity test

Model validity means that the model can accurately represent the actual system, and all simulation models need to be tested for validity. There are many ways to test the SD model, but due to the complexity of the research problem, the model cannot be connected with the real data. Therefore, the verification of the model focuses on whether the model is consistent with the actual trend, in other words, whether the model can produce "reasonable" results.

A.  System boundary inspection

One of the keys to the feasibility of the SD model is whether the model has a clear system boundary. The variables and time span in the model will affect the system boundary. Therefore, it is necessary to test the system boundary of the important conceptual variables in the model. The research object of this paper is the performance of medical-elderly care integrated institutions. The variables used in the model come from the existing research results. All the variables are core variables. The variables with less impact on the system are discarded, and the equations and loop settings are systematically revised after consulting experts in relevant fields. Therefore, the SD model established in this paper is effective.

B. Fitting test

**Fig. 3:**
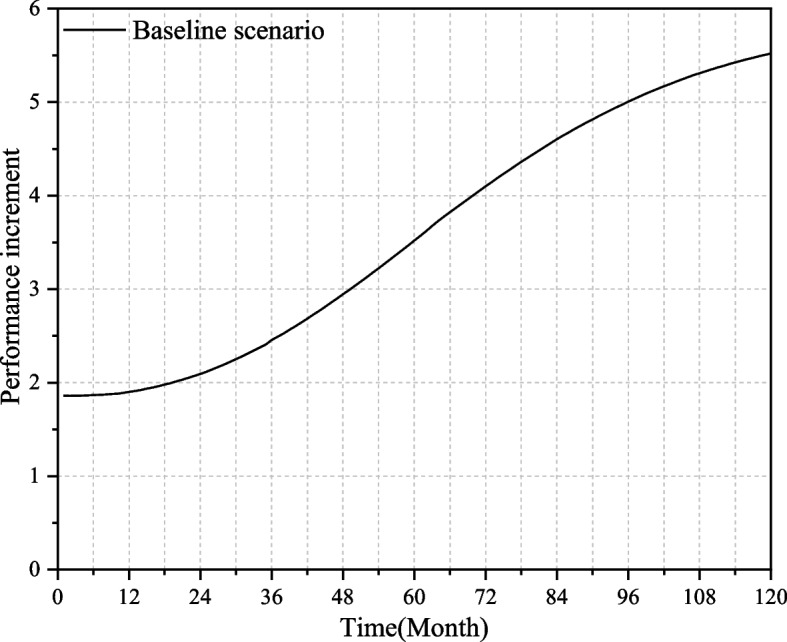
Performance increment of medical-elderly care integrated institutions

The development trend of China's medical-elderly care integration model has not changed much in the short term, and the institutions can form a good cooperation mechanism after a certain running-in period. Therefore, the simulation cycle is set to 10 years (120 months). The model is tested by using Vensim PLE software to verify whether the behavior and trend of each variable can show the actual development trend and law of the performance of medical-elderly care integrated institutions. As can be seen from Fig. 3, in the first two years, the performance increment of the institutions (referring to the performance level of the current month, while the institutional performance is the cumulative value of the performance increment) remained basically unchanged. After the running-in of elderly care institutions and medical institutions, the cooperation mechanism was gradually improved. From the third year, the performance increment of the institution began to increase rapidly, and after eight years, the performance increment tended to the level, indicating that the institution entered a stable operation state, which is in line with the performance changing trend in reality. Figure 3 also proves from another point of view that the system has been able to operate stably in 120 months, and the simulation time of 10 years set is reasonable.

III. Extreme condition test

**Fig. 4:**
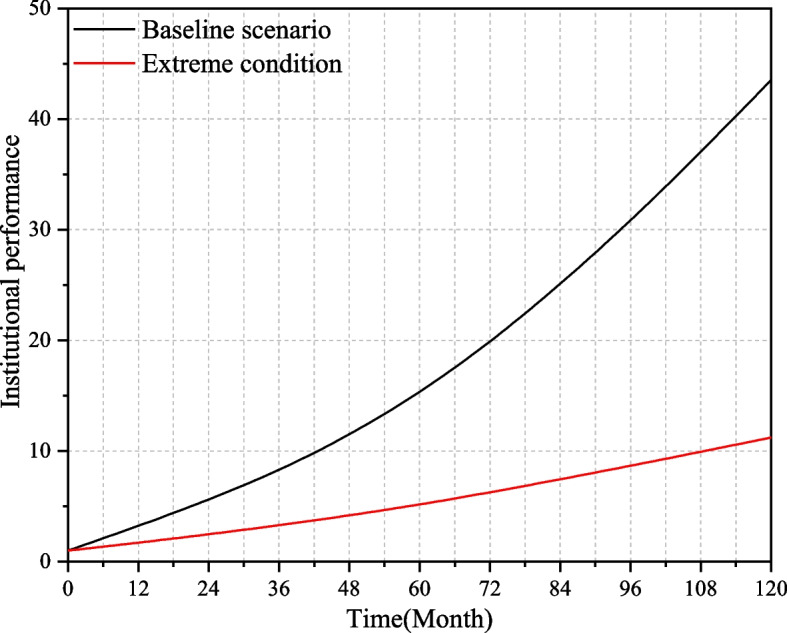
Institutional performance under the baseline scenario and extreme conditions

Take the performance level that can be achieved under the current technical conditions, investment capacity, and policy environment of the medical-elderly care integrated institutions as the baseline scenario. Put the key factors under the extreme conditions, that is, the value of relevant variables is set to 10% of the baseline scenario, and the results are shown in Fig. 4. Under extreme conditions, the performance improvement of the institution is very slow, and the performance level is always far lower than the baseline scenario, indicating that under the condition of lack of resource investment, the institutions can only rely on the original facilities to provide basic elderly care services, and cannot carry out the medical-elderly care integrated services. At this time the institution cannot obtain good benefits, which proves that the simulation results are in line with reality and that the equation setting in the model is feasible.

### Performance simulation under the baseline scenario

**Fig. 5:**
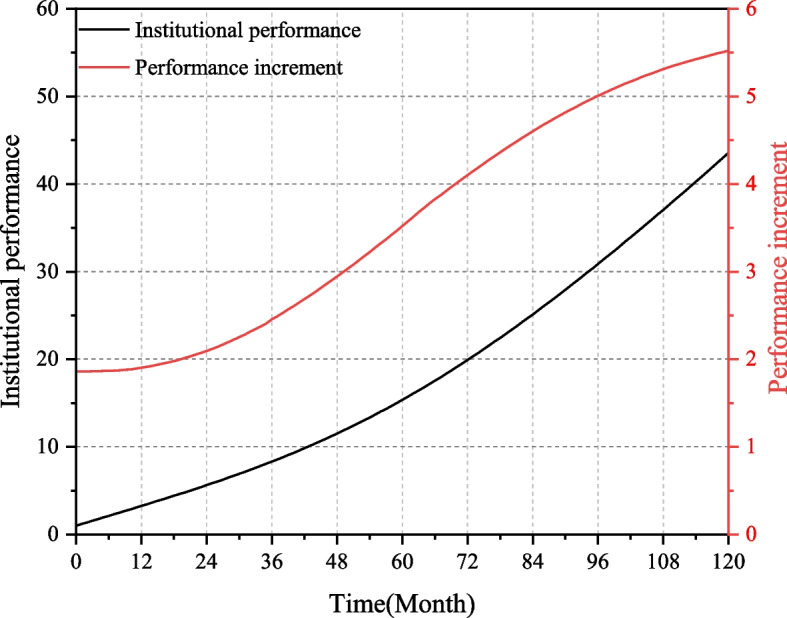
Institutional performance and performance increment under the baseline scenario

Under the baseline scenario, institutional performance and its increment are shown in Fig. 5. To reflect the changing trend of the curve clearly, this paper places institutional performance and performance increment in two coordinate systems. In Fig. 5, the change range of institutional performance level is [0,60], and the change range of performance increment is [0,6].

From the simulation results, it can be seen that the performance of medical-elderly care integrated institutions shows an upward trend, and the growth rate of institutional performance first increases and then stabilizes. On the one hand, as the input resources are gradually transformed into the output, the service quality and service ability of the institutions are improved, and the growth rate of institutional performance in the early stage is accelerating. On the other hand, the government's capital investment, quality supervision, and talent training have gradually played a role in promoting the development of institutions. In the latter stage, the incremental value of performance has gradually stabilizes at about 5.5, so the growth rate of institutional performance also tends to be stable.

### Single-factor sensitivity analysis

Analyze the changes in institutional performance under the change of three key factors: element input, service level and policy support. Therefore, the model simulates the following three scenarios based on the baseline scenario.

**Fig. 6:**
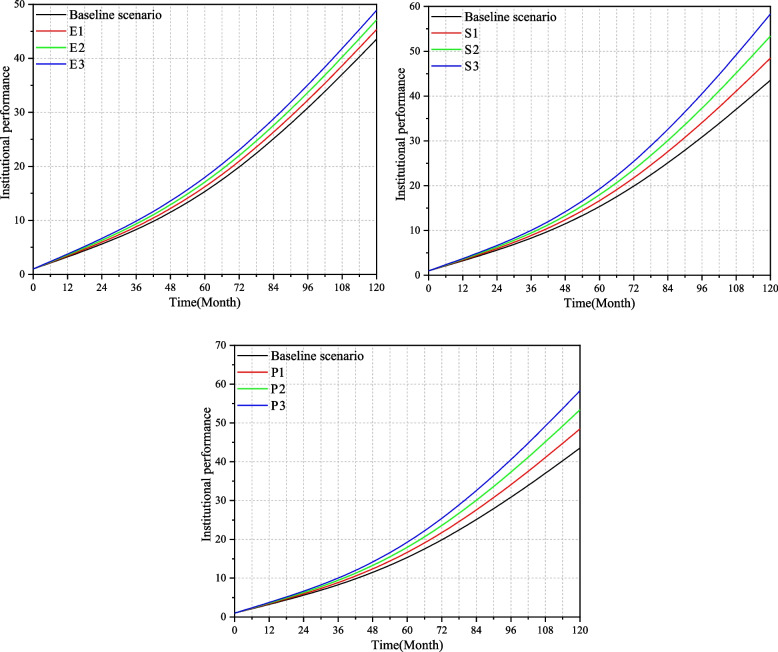
**(1)** Element input sensitivity analysis. (**2**) Service level sensitivity analysis. (**3**) Policy support sensitivity analysis

A. Simulate the changes of institutional performance when the system input increases, and increase the "Element input" variables to 20%, 40%, and 60% respectively on the basis of the baseline scenario to obtain E1, E2, and E3. See Fig. 6 (1) for the sensitivity analysis of element input.
B. Simulate the change of institutional performance when increasing the content of services and improving the service quality. Increase the "Service level" variable to 20%, 40%, and 60% respectively based on the baseline scenario to obtain S1, S2, and S3. See Fig. 6(2) for the sensitivity analysis of service level.
C. Simulate the change of institutional performance when the government increases its support for medical-elderly care integrated institutions, and increases the "Policy support" variable to 20%, 40%, and 60% based on the baseline scenario to obtain P1, P2, and P3 respectively. The sensitivity analysis of policy support is shown in Fig. 6(3).

From Fig. 6, it can be seen that policy support has the greatest impact on the performance of medical-elderly care integrated institutions, followed by service level, and the impact of institutional human and material resources input on institutional performance is relatively small. In order to more intuitively compare the influence of three key factors on institutional performance, this paper express the influence of key factors on institutional performance as "Institutional performance improvement rate", and the formula is:

1 $$\left\{\mathrm E-\mathrm{Improvement}\;\mathrm{rate}=\frac{\mathrm E3-\mathrm{Baseline}\;\mathrm{scenario}}{\mathrm{Baseline}\;\mathrm{scenario}}\right.$$

2 $$\left\{\mathrm S-\mathrm{Improvement}\;\mathrm{rate}=\frac{\mathrm S3-\mathrm{Baseline}\;\mathrm{scenario}}{\mathrm{Baseline}\;\mathrm{scenario}}\right.$$

3 $$\left\{\mathrm P-\mathrm{Improvement}\;\mathrm{rate}=\frac{\mathrm P3-\mathrm{Baseline}\;\mathrm{scenario}}{\mathrm{Baseline}\;\mathrm{scenario}}\right.$$

**Fig. 7:**
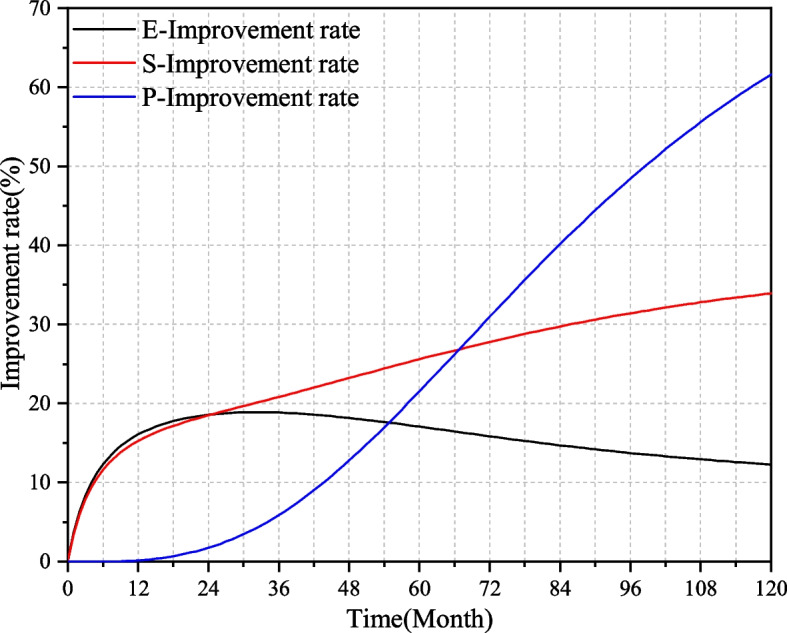
Improvement rate of three key factors on the institutional performance

The improvement rate of the three key factors on institutional performance is shown in Fig. 7. It can be found that in the first two years, the improvement rate of element input and service level increased rapidly, and then the two have different trends: the impact of element input on institutional performance increased rapidly in the early stage and then decreased gradually, while the impact of service level maintained a steady increase. Compared with the first two, the influence of policy support on institutional performance varies greatly in different periods. In the first three years, policy support has little impact on institutional performance, but later its influence increased rapidly until the tenth year. It can be found that although the government's support has been relatively slow to promote the development of medical-elderly care integrated institutions, it can gradually play a huge role over time.

### Multi-factor comprehensive simulation analysis

**Fig. 8:**
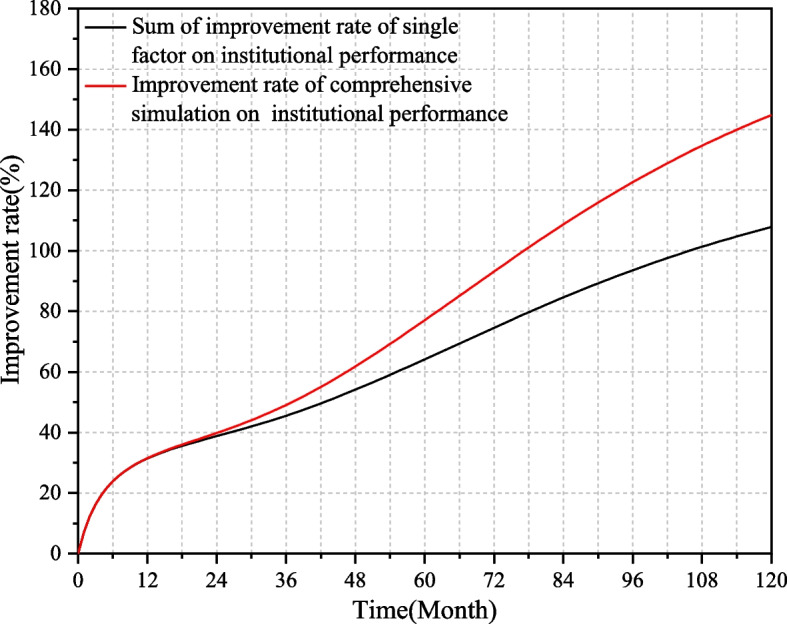
Comparison of improvement rate of single-factor and multi-factor simulation

Increase the values of the three key factors to 160% of the baseline scenario at the same time for multi-factor comprehensive simulation. This paper compare the sum of the improvement rate of single-factor with the improvement rates of comprehensive simulation. The results are shown in Fig. 8, which shows the emergent effect of the system, that is, the whole is greater than the sum of parts. From the third year of operation of the institution, the improvement rate on the institutional performance of the comprehensive simulation has always been greater than the sum of the improvement rate of the individual effects, and the gap between the two is increasing. In the 120th month, the sum of the improvement rate on the institutional performance of the individual effects is 108%, while the improvement rate of the comprehensive simulation is reaches 145%.

## Discussion

This paper constructs the performance evaluation SD model of medical-elderly care integrated institutions from three subsystems: System input, Service output, and Institutional satisfaction. The validity test results show that the model can well reflect the actual impact of key factors on institutional performance. The simulation results of the model show that the policy support level has the most significant impact on the performance of medical-elderly care integrated institutions. Government financial support, the supervision of institutional service quality, and the formulation of service standards, as well as increasing the training of professional elderly care service personnel, can significantly improve the service level of institutions and promote the development of institutions. The quality and diversity of medical-elderly care integrated services have the most direct impact on the satisfaction of the elderly. Improving the service quality and improving the personalized service level can make the elderly get a more comfortable old age life. The investment of institutional facilities and human resources are the foundation of institutional development, and the investment level of institutions has played an important role in the early stage of development. In addition, it should be noted that the internal investment level and service level of institutions are closely related to the policy support in the external environment. The impact of the comprehensive change of the key factors on the institutional performance is higher than the sum of the impact of the individual change of the factors, which indicates that the system has emergence attributes that cannot be explained by a single element [53].

SD is relatively mature in the research of institutional performance. This paper applies the SD method to the performance evaluation of medical-elderly care integrated institutions under the background of the Chinese model of medical-elderly care integration and explores the impact of key factors on the institutional performance by changing some parameter values. The model can be used to simplify the operating of the system and can be used as a reference for researchers in the real world [54]. It is worth noting that the simulation results of the SD model in this study are not predictions, but explore the potential role of institutional resource investment, service level, and government policy support on the changing trend of institutional performance and the development of medical-elderly care integrated institutions.

In addition, there are still several limitations to this paper. First of all, the application level of the Internet has been steadily improved in China, and perhaps the Internet penetration rate may reach 80% in a few years [52]. In the elderly care service industry, will the development of the Internet and the application of intelligent technology replace the work of some nursing staff [55], provide more diversified and personalized services, and reduce the demand for nurses staff [8]? In this context, should science and technology investment be included in the system and reduce the proportion of human investment? Secondly, the requirement of normalizing of COVID-19 epidemic prevention and control has forced people to change their lifestyles. More seriously, COVID-19 has a higher mortality rate for the elderly, especially the elderly with underlying diseases [56, 57]. For safety reasons, should the performance evaluation system of medical-elderly care integrated institutions take the epidemic prevention and control level into account? Finally, as the simplification of the real world, the model constructed in this paper will inevitably have incomplete and incorrect problems [58]. As mentioned earlier, the model is not tested with the data of empirical research results, so this will prompt us to carry out data collection and observation work in the future, so as to make more in-depth research and demonstration on the subject of this paper.

## Conclusion

Based on the above analysis, this paper believes that the strong support given by the Chinese government [59] has provided rich soil for the development of medical-elderly care integrated institutions, resulting in a sharp increase in the number of institutions in recent years. However, fundamentally, integrating the service resources of "medical care" and "elderly care" and providing more diversified integrated services to the elderly are the decisive factor for the good development of medical-elderly care integrated institutions. In the process of transformation, traditional elderly care institutions should pay attention to the system perspective. The institutional strategic planning should be combined with the national policy line, the level of institutional resource investment, the operation status of the institution, the demand characteristics of the elderly, and other factors, in order to make the best use of existing resources to improve the level of medical and elderly care integrated services. Policy support also plays an indispensable role in the development of medical-elderly care integrated institutions. In the process of promoting the mode of medical-elderly care integration, the government should actively perfect the talent training mechanism and improve the overall service level of the elderly care industry. At the same time, it should attract the participation of social capital to expand the investment of institutional resources and lay a more solid foundation for the long-term development of medical-elderly care integrated institutions.

## Data Availability

The datasets generated and/or analysed during the current study are available from the corresponding author on reasonable request.

## References

[1] Wang HC, Sheng JH (2018). Research Report on healthy city construction in China.

[2] Su K (2019). “Four measures simultaneously” to improve the satisfaction of medical care combined with elderly care service. People's Tribune.

[3] Yuan X, Jin N (2021). The basic point, focus and key points of the governance of the combination of medical care and nursing care model in China. J Hohai Univ.

[4] Li CY (2022). Practice exploration and promotion strategy of elderly care service combining medical care with elderly care – based on the observation of three typical pilot areas. Southwest Finance.

[5] World Health Organization (2012). Modern health care delivery systems, care coordination and the role of hospitals.

[6] Cheng QX, Feng ZY (2015). American PACE and its enlightenment on the combination of medical care and nursing care in Chinese communities. Medi Philos.

[7] Gori C, Fernández J, Wittenberg R (2015). Long-term care reforms in OECD countries.

[8] Chen SH, Huang YN, Xu YJ, Ji LP, Huang RD (2022). Research progress on the elderly care service model of medical and nursing integration in the context of "Internet plus". China Mod Med.

[9] Shaw S, Rosen R, Rumbold B (2011). An overview of integrated care in the NHS: what is integrated care.

[10] Department of Health and Social Care. Care Act facts-heets[EB/OL].(2018–10–23)[2020–05–10].http://www.gov.uk/government/publications/care-act-2014-part-1-factsheets/care-act-factsheets.

[11] Shaw L (2014). Program of all-inclusive care for the elderly: a comprehensive, cost-effective alternative for frail elderly individuals. N C Med J.

[12] MacAdam M (2015). PRISMA:program of research to integrate the services for the maintenance of autonomy. A system-level integration model in Quebec. Int J Integr Care.

[13] Dong EH, Bao Y, Liu W (2016). Summary of research on the linkage mode between community medical institutions and elderly care institutions under the background of "medical care integration". China Health Adm.

[14] Hao L, Yan C, Yang XK (2015). Evaluation index system of community elderly care service development based on 3C elements. Chin J Gerontol.

[15] Zhu M, Wang YY (2022). Typical cases, practical difficulties, and Countermeasures of medical care combined service model under the background of aging. Human Resour Soc Secur.

[16] Cui SY, Yang SW (2019). Research on "combination of medical care and nursing" from the perspective of healthy China. Dongyue Tribune.

[17] Li D, Li LP (2022). Research on the high-quality supply of community medical care and elderly care services. Acad J Zhongzhou.

[18] Zhu L, Yang XJ, Zhang Q, Wang FL, Zhang XL, Xing FM (2019). Research on the construction of service quality evaluation index system of community home-based elderly care center with the combination of medical care and nursing. Chin Gen Pract.

[19] Guo HY, Wang L, Peng JL, Xie H (2013). A qualitative study on the managers' experience of quality management in elderly care institutions. J Nurs Adm.

[20] Xiao XH, Huang ZM, Wu YF, Yan MQ, Li B (2019). Construction of service performance evaluation index system of medical and elderly care institutions. Chin Gen Pract.

[21] Xiong Y, Shu Y, Chen Y (2021). Research on system dynamics of strategic human resource management and innovation performance of alliance enterprises. Chinese Certified Public Accountant.

[22] Bianchi C, Bivona E, Cognata A, Ferrara P, Landi T, Ricci P (2010). Applying system dynamics to foster organizational change, accountability, and performance in the public sector: A case-based Italian perspective. Syst Res Behav Sci.

[23] Mudhafar A, Mohammed A, Konstantinos S. A system dynamics model of employees’ performance. Sustainability. 2020;12(16). 10.3390/su12166511.

[24] Federico C (2018). Supporting public sector management through simulation-based methods: a dynamic performance management approach. Int Rev Public Adm.

[25] Yadav N (2020). Application of system dynamics methodology in performance management system: a case study of Indian automotive firm. Int J Bus Perform Manag.

[26] Ghaffarzadegan N, Lyneis J, Richardson GP (2011). How small system dynamics models can help the public policy process. Syst Dyn Rev.

[27] Yuan HP, Shen LY, Hao JL, Lu WS (2011). A model for cost-benefit analysis of construction and demolition waste management throughout the waste chain. Resour Conserv Recycl.

[28] Xiong W, Yuan JF, Li Q, Skibniewski MJ (2015). Performance objective-based dynamic adjustment model to balance the stakeholders’ satisfaction in PPP projects. Civ Eng Manag.

[29] Li J (2011). Investigation on service quality and its influencing factors of urban elderly care institutions. J Soc Work.

[30] Xie JF, Yu JZ, Shao MH, Cheng YW (2014). Establishment of community nursing quality evaluation index system. Shanghai Nurs.

[31] Zhang ZY, Zhao J, Shi YQ (2013). Innovative supply chain model of elderly care service: performance evaluation and optimization strategy – Based on the survey of Liwan District, Guangzhou. Commercial Res.

[32] Li JD (2018). Construction and empirical research on the evaluation index system of the "combination of medical care and elderly care" elderly care model. Shandong Acad Med Sci.

[33] Zhu FM (2019). Cause analysis of "low occupancy rate" of private elderly care institutions: evidence from city and county levels. Population J.

[34] Yu SK, Liu YH, Hu LD (2014). Establishment and application effect of nursing service quality evaluation system. Heilongjiang Med J.

[35] Wang LJ, Feng Y, Wang C (2017). Research on service quality evaluation of elderly care institutions. Popul Dev.

[36] Xu Q, Chang XL (2018). Research on performance evaluation of medical and nursing combined elderly care service in elderly care institutions in Qingdao. J Qingdao Univ Sci Technol.

[37] Zhang XY, Mei Q (2012). Research on performance evaluation index system of community home-based elderly care service. Statistics Decision.

[38] Liu YJ (2020). Research on service performance evaluation of medical and elderly care institutions. Shandong Univ Arch.

[39] Liu GH, Fan YQ, Liu ZS, Fan LQ, Zhong RG (2020). How does the combination of medical care and elderly care affect the service efficiency of private elderly care institutions-Evidence from Beijing. Manag Rev.

[40] Fang JY, Zhang HC, Chen WQ, Shen Y, Wang SS, Ju CH, Guo Q (2020). Study on the construction of health service quality index system of medical and nursing combined elderly care institutions. Chin J Health Policy.

[41] Zhang LW, Zeng YB, Fang Y (2019). Construction and application of comprehensive evaluation index system for service quality of elderly care institutions. Chin J Health Stat.

[42] Yin HR, Yuan H, Peng X, Li WT, An LB (2016). Construction of evaluation index system for elderly care service ability of urban non-profit elderly care institutions. Chi J Gerontol.

[43] Zhang YY, Han NN (2019). Research on service quality evaluation of medical and nursing combined elderly care institutions. Health Econ Res.

[44] Zhang L (2020). Research on performance evaluation of public pension institutions under the mode of medical care integration. Contemp Account.

[45] Li HL (2020). Research on performance evaluation index system of medical and nursing institutions in Gansu Province. Lanzhou Univ.

[46] Fang Y, Chen Q, Lei Y (2018). Performance evaluation of medical care combined with home-based elderly care service. Manage Observ.

[47] Yang YL. Research on the performance of private elderly care institutions under the mode of combination of medical care and elderly care. Master's Thesis. Zhengzhou University, Zhengzhou, China. 2019. https://kns.cnki.net/KCMS/detail/detail.aspx?dbname=CMFD201902&filename=1019103591.nh.

[48] Zhu BS, Qiao XC (2018). Study on Influencing Factors of the occupancy rate of elderly care institutions in Beijing – Based on the survey data of elderly care service facilities in Beijing. World Survey Res.

[49] Tian Y, Cui SY, Yang SW (2018). Research on the implementation effect of supporting policies for elderly care institutions – Based on the investigation and analysis of 45 elderly care institutions in Shandong Province. J Shandong Univ.

[50] Sun XL, Han ZY (2022). Research on the content of medical care integration policy from the perspective of policy tools – Based on the analysis of Nanjing policy text from 2006 to 2020. Chin Health Serv Manag.

[51] An C, Kai W (2020). System Dynamics Analysis of Innovation Performance of High-tech Enterprises-Based on the Perspective of Knowledge Spillover. Chin J Syst Sci.

[52] Liu J, Jia HJ (2012). Prediction of Internet penetration in China based on innovation diffusion theory.

[53] Luke DA, Stamatakis KA (2012). Systems science methods in public health: dynamics, networks, and agents. Annu Rev Public Health.

[54] Geoff C, David E (2000). The validation of commercial system dynamics models. Syst Dyn Rev.

[55] Kong S (2022). Preliminary study on building a new era of Chinese characteristics medical and nursing integration model. People's Tribune.

[56] Yang GX, You HM, Hu CG (2020). Rethinking the planning of community health care and elderly care services in the context of epidemic prevention and control [J]. Urban Problems.

[57] Du P, An RX (2021). The impact of the COVID-19 on health services for the elderly and Its Enlightenment. J Hebei Univ.

[58] Joachim. The mystery of job performance: a system dynamics model of human behavior. 2014. https://proceedings.systemdynamics.org/2014/proceed/papers/P1124.pdf.

[59] Sun JJ, Tian JY (2020). Analysis of China's policy of combining medical care with nursing care from the perspective of new health aging. China Sport Sci Technol.

